# Developmental Profiling of Dietary Carbohydrate Digestion in Piglets

**DOI:** 10.3389/fmicb.2022.896660

**Published:** 2022-04-29

**Authors:** Xiaoqian Gao, Bing Yu, Jie Yu, Xiangbing Mao, Zhiqing Huang, Yuheng Luo, Junqiu Luo, Ping Zheng, Hui Yan, Jun He, Daiwen Chen

**Affiliations:** Key Laboratory of Animal Disease-Resistance Nutrition, Ministry of Education, Institute of Animal Nutrition, Sichuan Agricultural University, Chengdu, China

**Keywords:** carbohydrate, microbiome, birth, weaning, piglet

## Abstract

Carbohydrates are the main source of energy in the diet, accounting for the largest proportion in the diets of humans and monogastric animals. Although recent progress has been made in the study of intestinal carbohydrate digestion in piglets, there is a lack of comprehensive study on the dynamic changes in intestinal carbohydrate digestion with age in the early growth stage of piglets. To fill in this gap of knowledge, we collected samples of the small intestine, pancreatic tissues, and colonic digesta from 42 piglets during newborn [day (d) 0], lactation (d 7, 14), weaning (d 21), and nursery (d 28, 35, and 42) stages. Intestinal and pancreatic tissues and colonic digesta were collected at necropsy and analyzed for morphology, digestive enzyme activities, short-chain fatty acids (SCFA), and microbial abundance. Villus height reached a maximum at 1 week (d 7) in the duodenum and jejunum (*P* <0.01), and a higher ratio of villus height to crypt depth and lactase activity were observed on d 0 and 7 (*P* < 0.001) compared to other ages. However, the sucrase and maltase activities were increased with piglets' age. Similar activities of sucrase and maltase were found in the small intestine. In addition, amylase, lipase, and protease activities were assayed in the pancreas. The activity of amylase increased with age, while lipase and protease decreased gradually from birth to weaning (d 21, 28) and then increased after weaning (d 35, 42). Compared with d 0, d 42 increased the abundance of *Firmicutes* and *Bacteroidetes* with a higher concentration of total SCFA (*P* < 0.001) and decreased the abundance of *Proteobacteria*, but weaning (d 21, 28) increased the abundance of *Proteobacteria* in the colon. These results indicate that with the increase in piglet age, the carbohydrate digestive function gradually increased, but weaning hindered the development of intestinal function. These results provide us with new insights into the healthy development of piglets' intestines, which may help us to better regulate the physiological health of piglets in the future.

## Introduction

In recent years, the impact of dietary carbohydrates on health has become one of the main focuses in the field of public health. As one of the three essential nutrients, carbohydrates provide about 60% of energy in the Asian diet (Cui and Dibley, 2012). Therefore, it plays an important role in the nutrition supply of human or monogastric animals during lactation and nursery stages. During lactation, lactase is the main source of energy; after weaning, the starch in food is the main source of energy (Corring et al., 1978). However, due to an immature gastrointestinal tract, young children may experience temporary malabsorption when consuming starchy foods, which may lead to disease in serious cases (Lin, 2018; Shulman, 2018). In contrast to the slow weaning process of human infants, piglets in commercial animal production experienced a huge shift from high-fat, low-carbohydrate breast milk to high-carbohydrate, and low-fat solid feed in 21–28 days of life. This change will lead to diarrhea and intestinal damage in piglets and then affect growth performance (Xiong et al., 2019). Therefore, this may bring an economic burden on the swine industry (Dou et al., 2017). Over the last decade, animal nutritionists have improved the overall health of weaned piglets by continuously optimizing feed formulations to meet the needs of weaned piglets and exploring different nutritional factors or management (Xiong et al., 2019). Studies have found that the gut of mammals is home to trillions of microbes that play a crucial role in nutrient absorption and metabolism (Hu et al., 2016). Intestinal microbial composition and ecological succession are determined by some complex internal and external factors, such as age, weaning, and diet (Kim et al., 2012; Yatsunenko et al., 2012). Because microorganisms reproduce in the host and change with age, it is very important to study the community structure of intestinal microorganisms (Green et al., 2006; Kim et al., 2012; Krajmalnik-Brown et al., 2012). In addition, the role of microbiota in health has attracted more and more attention (Ramayo-Caldas et al., 2016). Miniature piglets are similar to humans in physiological, anatomical, and endocrine systems, especially in infancy. Therefore, it is of great significance to study the changes in intestinal digestive enzymes and microbiota in miniature weaned piglets (Hu et al., 2016). Few studies have examined changes in intestinal digestive enzymes and microbes in piglets at different time periods, and there are currently no longitudinal studies that follow the correlation between digestive enzymes and microbes in piglets from birth to nursery.

In this study, we followed the changes in the digestive function, microbial communities, and the correlation between intestinal microorganisms and digestive enzymes and microbial metabolites in piglets from birth through weaning, up to 6 weeks of age. More importantly, this experiment covered the effects of diet, weaning, and age on the health of piglets. It is hoped that the results of this experiment will contribute to a deeper understanding of the changes in the digestive physiology of piglets and pave the way for the formulation of better nutritional strategies to promote the overall health of infancy and piglets.

## Materials and Methods

### Ethics Statement

The experimental protocols used in the present study were approved by the Sichuan Agricultural University Institutional Animal Care and Use Committee No. 69130079.

### Animals and Experimental Treatments

Six multiparous sows were chosen for this study, with similar parity and health status. Upon delivery, the neonatal piglets were cohoused with sows by litter. Before weaning at 21 days of age, neonatal piglets are allowed to feed freely and, as far as we know, they do not eat sow feed. After weaning, piglets were removed from the sow and transferred to separate housing and fed *ad libitum* with the same basal diet (maize–soybean meal diet) formulated by the National Research Council (NRC, 2012) (Table 1). Consequently, a total of 42 piglets (Duroc × Landrace × Yorkshire) with similar body weights were studied. The piglets had no access to antibiotics.

**Table 1:** Ingredients and chemical composition of experimental diets (as-fed basis).

**Ingredients**	**Content, %**	**Nutrient composition^c^**	**Content, %**
Corn (7.8% crude protein)	24.8	Digestible energy, Mcal/kg	3.54
Extruded corn (7.8% crude protein)	28.00	Crude protein	19.69
Extruded soya bean	7.00	Calcium	0.80
Soybean meal, dehulled	14.00	Available phosphorus	0.36
Fish meal (62.5% crude protein)	5.00	Lysine	1.35
Whey powder	9.00	Methionine	0.39
Soy protein concentrate	5.00	Methionine + Cysteine	0.70
Soybean oil	3.00	Threonine	0.81
Glucose	2.00	Tryptophan	0.23
NaCl	0.35		
Limestone	0.93		
Dicalcium phosphate	0.26		
L-Lysine–HCl (78%)	0.21		
DL-Methionine	0.05		
Chloride choline	0.15		
Vitamin premix^a^	0.05		
Mineral premix^b^	0.20		
Total	100		

a *The vitamin premix provided the following per kg of diets: 6,000 IU vitamin (V) A, 3,000 IU VD3, 24 mg VE, 3 mg VK3, 1.5 mg VB1, 6 mg VB2, 3 mg VB6, 0.02 mg VB12, 14 mg niacin, 15 mg pantothenic acid, 1.2 mg folic acid, and0.15 mg biotin*.

b *The mineral premix provided the following per kg of diets: 100 mg Fe, 10 mg Cu, 80 mg Zn, 4 mg Mn, 0.30 mg I, and 0.35 mg Se*.

c *Values are calculated composition*.

### Tissue Collection and Processing

At Days 0, 7, 14, 21, 28, 35, and 42 after birth, one piglet per group from each of the six litters was euthanized with intravenous injection of sodium pentobarbital (200 mg per kg, BW). After the slaughter, the abdominal cavity had been opened. The intestinal segments of the distal duodenum, mid-jejunum, and ileum were flushed gently with ice-cold phosphate-buffered saline (PBS) and then fixed in 4% formaldehyde-phosphate buffer for intestinal histology. Tissue samples of the small intestine, pancreatic, and colonic contents were immediately frozen in liquid nitrogen and then stored at −80°C for further analysis.

### Intestinal Morphology

Intestinal morphology was measured using standard procedures (Pluske et al., 1996). In brief, fixed samples (duodenum, jejunum, and ileum) were dehydrated and embedded in paraffin, sectioned (5 μm thickness), and stained with hematoxylin and eosin (H&E). The specimens were examined using an Eclipse Ci-L microscope at 40 × magnification. A minimum of 10 well-oriented villi and crypts from each section were measured, and the ratio of villus height to crypt depth was calculated. The villus height and crypt depth were measured and analyzed using an Image-pro plus 6.0 (Media Cybernetics, Inc., Rockville, MD, USA).

### Digestive Enzyme Activities

The activity of brush border enzymes was determined in intestinal (duodenum, jejunum, and ileum) tissue, and trypsin, chymotrypsin, lipase, and amylase activities were determined in the pancreatic tissue. After thawing, 0.3–0.9 g of tissue was homogenized with ice-cold physiological saline and centrifuged for 10 min at 2,500 g at 4°C. The supernatant was collected for the determination of digestive enzyme activities and total protein. Total protein content was measured using the Bradford brilliant blue method (Nanjing Jiancheng Bioengineering Institute, Nanjing, China). The activities of lactase, maltase, and sucrase were measured by kit (Nanjing Jiancheng Bioengineering Institute, Nanjing, China). Except for lipase and chymotrypsin, enzymatic activity was expressed as nanomoles of substrate hydrolysis per minute per gram of protein (U/g protein). For other enzymes, the enzymatic activity was expressed as nanomoles of substrate hydrolyzed per minute per mg protein (U/mg protein).

### Short-Chain Fatty Acids

The SCFA concentrations in the colonic digesta were analyzed according to the method described by Porter and Murray (2001). In brief, 0.7 g of colonic digesta was put into a 2 ml centrifuge tube with 1.5-ml distilled water, then incubated on ice for 30 min, and mixed and centrifuged (15,000 rpm) at 4°C for 15 min. The supernatant (1 ml) was transferred into centrifuge tubes (2 ml) and mixed with 0.2 ml of metaphosphoric acid and 23.3 μl of crotonic acid. After 30 min at 4°C, the tubes were centrifuged (15,000 rpm) again at 4°C for 10 min. Then, 300 μl supernatant was transferred to another sterile tube, mixed with 900 μl methanol, and homogenized. After this, the mixture was centrifuged (1,000 rpm) at 4°C for 5 min. The supernatant was obtained and then filtered using a 0.22-μm nylon membrane filter (Millipore, Bedford, OH, USA). Finally, aliquots of the supernatant (1 μl) were injected into a gas chromatographic system (VARIAN CP-3800, Varian, Palo Alto, CA, America) to separate and quantify the SCFA.

### DNA Extraction and Illumina MiSeq

Total genomic DNA from the individual samples of colonic digesta was extracted using an E.Z.N.A Stool DNA Kit (Omega Bio-Tek, Doraville, GA) according to the manufacturer's instructions. The V3–V4 hypervariable regions of 16S rRNA were PCR-amplified from microbial genome DNA which was harvested from colonic digesta samples with the forward primer 341F (CCTACGGGNGGCWGCAG) and reverse primer 806R (GACTACHVGGGTATCTAATCC). The amplification mix contained 2 × Hieff^®^ Robust PCR Master Mix (dNTP and Mg^2+^) (Yeasen Co. Ltd., Shanghai, China), 1 μl of each primer, 10–20 ng of PCR products, and 9–12 μl H_2_O in a reaction volume of 30 μl. The PCR program initially started with 94°C for 3 min, followed by 25 cycles of 94°C for 30 s, 54°C for 20 s, and 65°C for 30 s, and then followed by a single final extension step at 72°C for 10 min. The PCR reaction system which was used to add a specific tag sequence was 30 μl, containing 2 × Hieff^®^ Robust PCR Master Mix (dNTP and Mg^2+^) (Yeasen Co. Ltd., Shanghai, China), 1 μl of each primer, 20–30 ng of PCR products, and 9–12 μl of H_2_O. The PCR conditions were 95°C for 3 min, followed by five cycles of 95°C for 20 s, 55°C for 20 s, and 72°C for 30 s, and then followed by a single final extension step at 72°C for 5 min. PCR product was excised from a 2% agarose gel, purified by Hieff NGS™ DNA Selection Beads (Yeasen Co. Ltd., Shanghai, China), and quantified by a Qubit 3.0 fluorometer (Invitrogen, USA). Library construction and Illumina MiSeq sequencing were carried out in Sangon Biotech (Shanghai) Co., Ltd. The information on DNA sequences was analyzed by QIIME software (Caporaso et al., 2010).

### Statistical Analysis

The data in the present study were analyzed by IBM SPSS 23.0 (Chicago, IL, USA) and expressed as means ± SEM. All parameters were assessed using each slaughtered piglet as an experimental unit. The data were evaluated by one-way ANOVA with Duncan's *post-hoc* test. A value of *P* < 0.05 was used to indicate statistical significance, whereas a *P*-value between 0.05 and 0.10 was considered to indicate a trend toward significance.

## Results

### Small Intestinal Morphology

**Figure 1:**
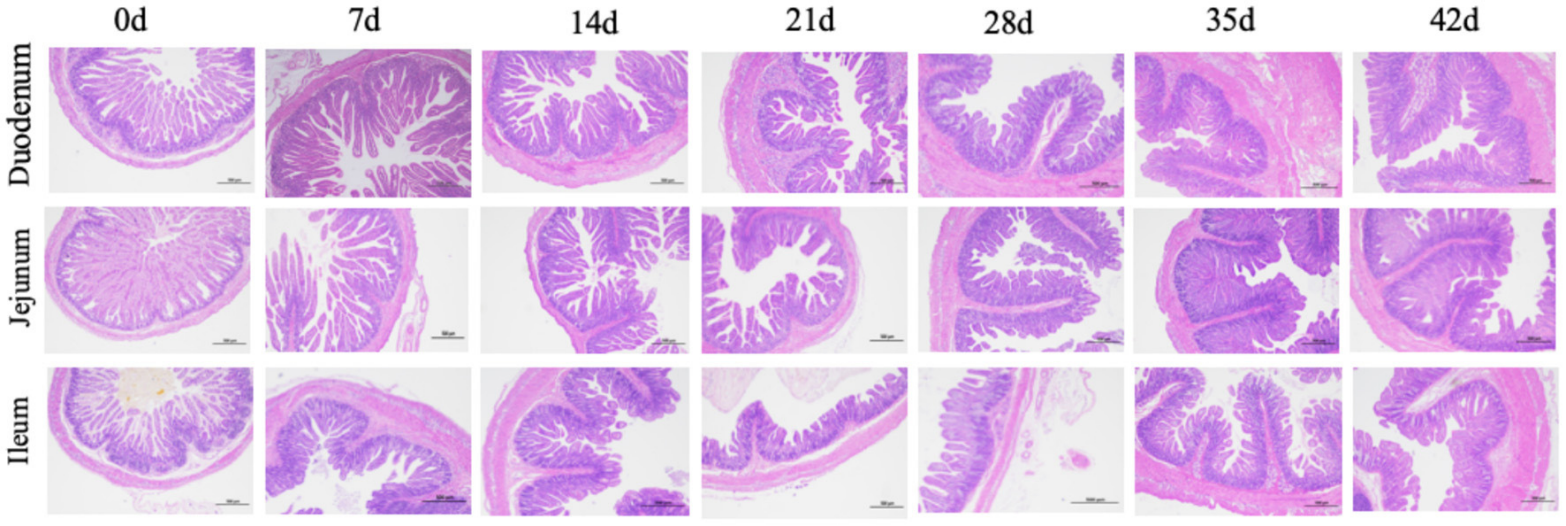
Histological evaluation of small intestine tissue in piglets with increasing age.

The small intestinal morphology of the piglets is shown in Table 2 and Figure 1. The results showed that in the duodenum, villus height, and villus/crypt ratio increased from birth to lactation, decreased during weaning, and then began to recover at 35–42 d (*P* < 0.001). In the jejunum, crypt depth increased at each step from birth to nursery with increasing piglet age (*P* < 0.001), while in the ileum villus height gradually decreased from birth to weaning and began to recover at 35–42 d (*P* = 0.008).

**Table 2:** Effect of piglet age on intestinal morphology.

**Item**	**0d**	**7d**	**14d**	**21d**	**28d**	**35d**	**42d**	**Pooled SEM**	***P*** **-value**
**Duodenum**									
Villus height, μm	319.93^bc^	429.47^a^	465.88^a^	339.88^b^	200.13^d^	255.38^cd^	275.16^bc^	15.96	<0.001
Crypt depth, μm	103.99	124.17	120.48	143.77	123.55	117.51	129.91	3.61	0.109
Villus height/crypt depth	3.12^bc^	3.52^ab^	3.89^a^	2.48^cd^	1.61^e^	2.22^de^	2.01^de^	0.15	<0.001
**Jejunum**									
Villus height, μm	472.73^ab^	533.51^a^	475.65^ab^	353.74^bc^	269.97^c^	317.40^bc^	347.03^bc^	22.88	0.007
Crypt depth, μm	80.20^d^	100.85^cd^	105.37^bc^	125.04^ab^	136.10^ab^	149.79^ab^	153.37^a^	6.47	0.010
Villus height/crypt depth	6.03^a^	5.38^ab^	4.84^ab^	3.45^bc^	2.05^c^	2.23^c^	2.44^c^	0.34	<0.001
**Ileum**									
Villus height, μm	447.32^a^	349.62^ab^	302.02^b^	273.03^b^	251.63^b^	340.35^ab^	360.54^ab^	15.37	0.008
Crypt depth, μm	81.25^b^	105.72^ab^	109.00^ab^	113.86^ab^	138.46^a^	116.55^a^	118.51^a^	4.48	0.037
Villus height/crypt depth	5.74^a^	3.34^b^	3.10^b^	2.72^b^	1.83 ^b^	3.01^b^	3.14^b^	0.26	0.001

*Mean values with their standard errors*.

a, b, c, d, e *Mean values within a row with different superscript letters were significantly different (P < 0.05)*.

### Digestive Enzyme Activity in the Intestine and Pancreas

**Figure 2:**
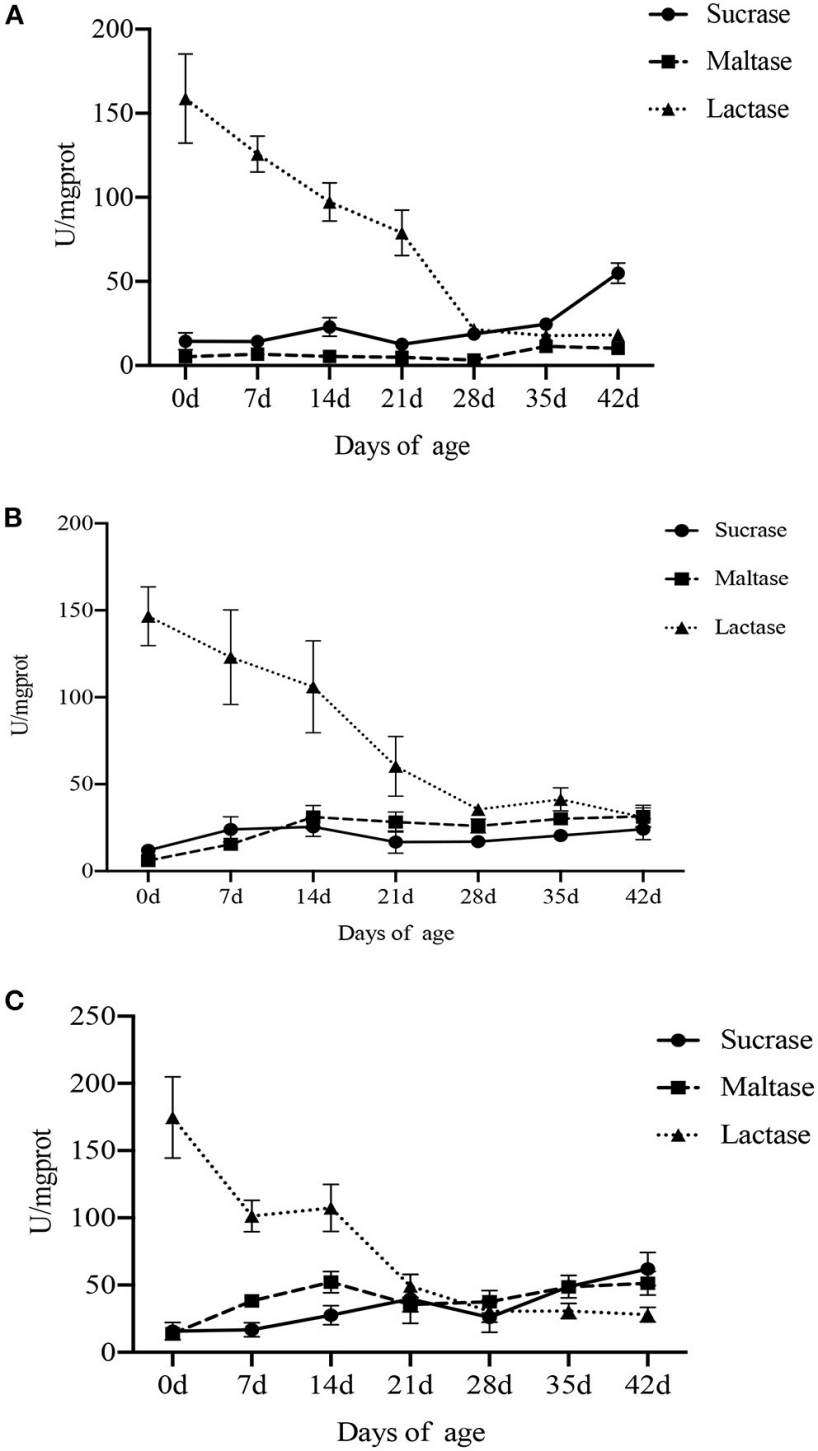
Effect of piglet age on intestinal disaccharidase activity. **(A)** Duodenum. **(B)** Jejunum. **(C)** Ileum. Values are expressed as means ± SEM.

**Figure 3:**
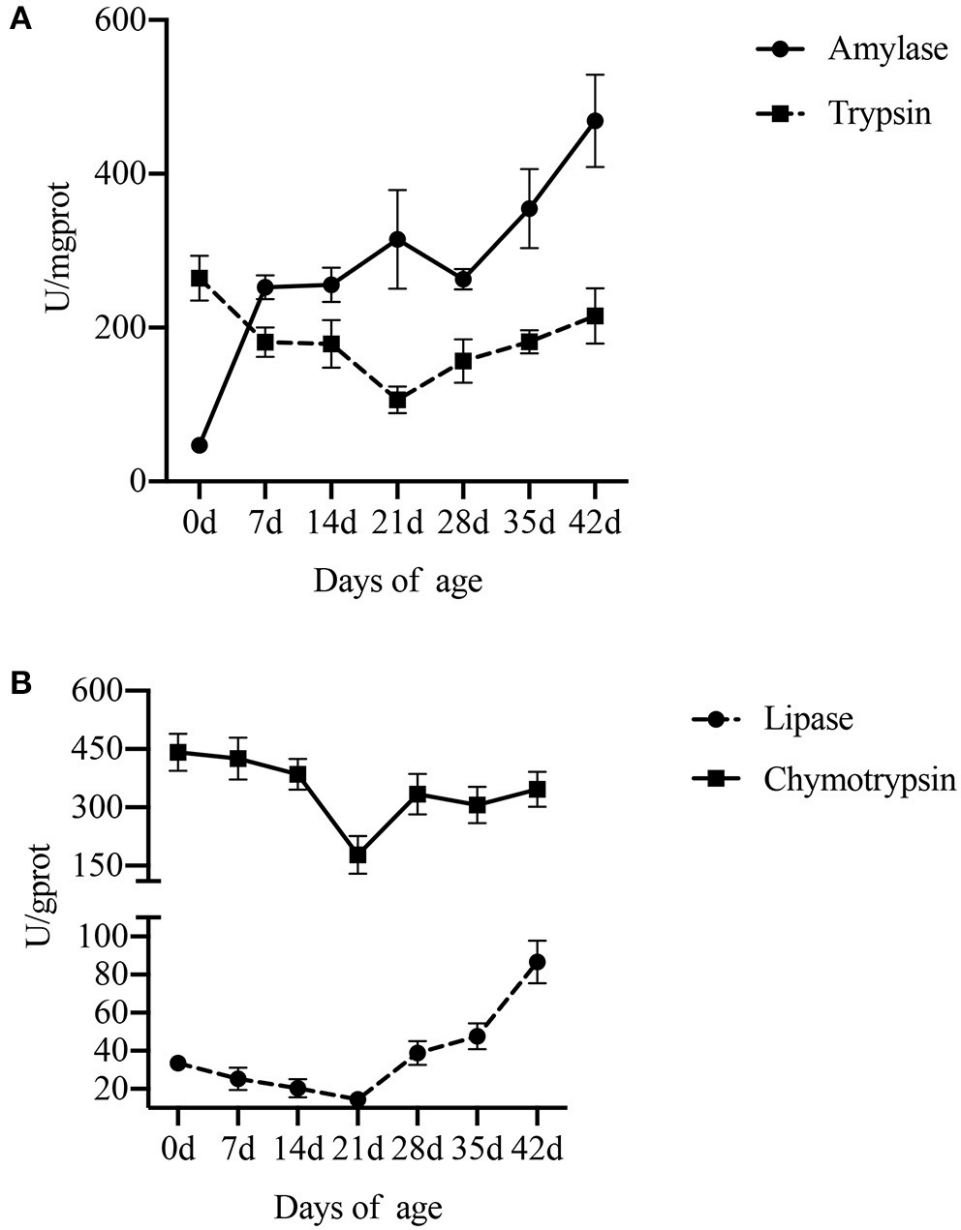
Effect of piglet age on pancreatic digestive enzyme activity. **(A)** Amylase and trypsin. **(B)** Lipase and chymotrypsin. Values are expressed as means ± SEM.

The effect of age on digestive enzyme activity in the small intestine and pancreas is shown in Figures 2, 3 (Supplementary Tables S1, S2). Compared with lactation and weaning, piglets in the nursery period (35–42 d) showed higher sucrase and maltase activities, and there was no significant difference between lactation and weaning. However, the activity of lactase was the highest at birth and gradually decreased with the increase in age, especially during weaning (*P* < 0.001).

Compared with lactation and weaning, piglets at birth had higher activities of trypsin (*P* = 0.018) and chymotrypsin (*P* = 0.010) in the pancreas. The activity of pancreatic lipase in piglets was low either during lactation or weaning and increased with the age after weaning (*P* < 0.001). As for amylase, the activity increased with the piglet age, especially in lactation (*P* < 0.001).

### The Concentration of Short-Chain Fatty Acids in Colonic Digesta

Table 3 shows the concentration of SCFA determined in the colonic digesta. At 42 d, we found significantly higher concentrations of acetate, propionate, butyrate, and total SCFA than those during lactation and weaning (*P* < 0.001). However, the concentration of SCFA was low at birth and weaning. In general, the concentration of SCFA increased with the age of piglets from birth to nursery (*P* < 0.001), but decreased during weaning and began to recover after weaning.

**Table 3:** Effect of piglet age on the yield of short-chain fatty acids (μmol/g of wet digesta) in colonic digesta.

**Item**	**0d**	**7d**	**14d**	**21d**	**28d**	**35d**	**42d**	**Pooled SEM**	***P*** **-value**
Acetate	2.15^d^	14.42^c^	17.52^c^	13.10^c^	18.78^bc^	24.42^b^	36.64^a^	1.76	<0.001
Propionate	1.20^d^	4.07^cd^	7.00^bc^	3.52^cd^	6.67^bc^	9.42^b^	17.25^a^	0.89	<0.001
Butyrate	0.48^c^	2.08^bc^	3.28^b^	1.66^bc^	1.37^bc^	3.10^b^	7.69^a^	0.45	<0.001
Isobutyrate	0.26^c^	0.55^c^	0.72^bc^	0.48^c^	0.57^bc^	1.07^ab^	1.46^a^	0.08	<0.001
Isovalerate	0.38^c^	0.73^bc^	1.05^bc^	0.71^bc^	0.66^bc^	1.23^bc^	1.99^a^	0.11	<0.001
Valerate	0.24^b^	1.11^ab^	0.73^ab^	0.46^b^	0.34^b^	0.51^b^	1.79^a^	0.15	0.048
Total short-chain fatty acids	3.80^d^	22.96^c^	30.30^bc^	19.94^c^	28.39^bc^	39.74^b^	66.82^a^	3.24	<0.001

*Mean values with their standard errors*.

a, b, c, d *Mean values within a row with different superscript letters were significantly different (P < 0.05)*.

### Bacterial Composition and Diversity

**Figure 4:**
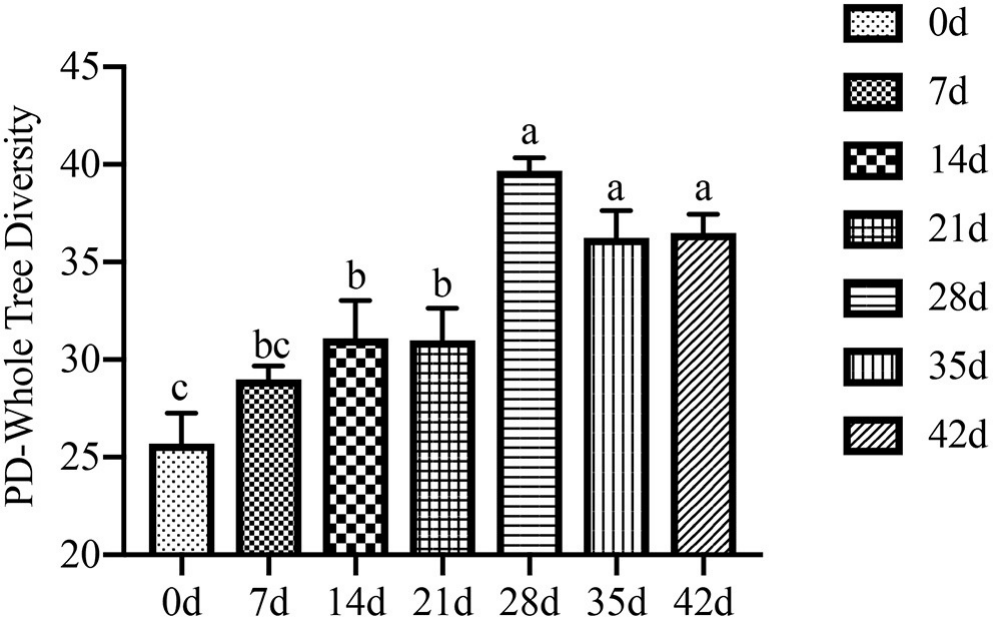
Effect of piglet age on colonic chyme α-diversity (phylogenetic distance, PD). Values are means with standard errors represented by vertical bars. a, b, c Mean values with unlike letters were significantly different within a cluster of bars, not across the clusters of bars (*P* < 0.05).

**Figure 5:**
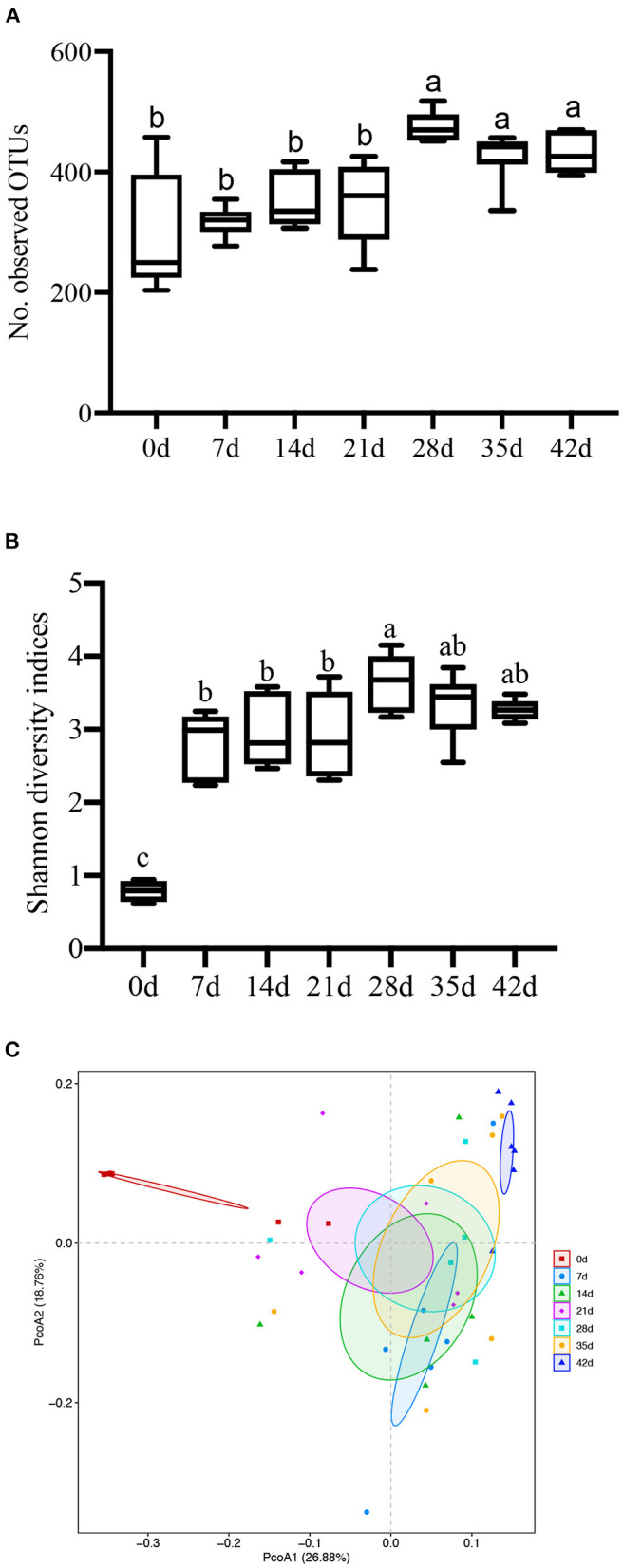
Effect of piglet age on α- and β-diversity of microbial communities in colonic digesta. **(A)** Bacterial α-diversity determined by no. of observed OTUs. **(B)** Bacterial α-diversity determined by the Shannon index. **(C)** Scatter plot from PCoA, based on weighted UniFrac distance in bacterial communities (0, 7, 14, 21, 28, 35, and 42 days after birth). Different letters above the bars denote a significantly different α-diversity index among groups.

In this study, an average of 59,433 clean tags was obtained for each group, and the length of the sequences ranged between 415 and 426 bp. α-Diversity analysis showed a sharp contrast among newborn, lactating, and nursery piglets (*P* < 0.001, Figure 4), and when examined over time, a gradual increase in the α-diversity (phylogenetic distance, PD) from 0 to 21 d is observed. This trend stabilized after 21 d until other time points in the study, but the differences between litters were not significant (*P* > 0.05). Furthermore, 3 weeks after weaning (28–42 d), the colonic digesta microbiota appeared to be more diverse and had greater evenness than that of lactation and weaning, according to the Shannon index (*P* = 0.009, Figure 5A) and observed OTUs (*P* < 0.001, Figure 5B). This trend stabilized after 28 d to other time points studied, but differences between litters were not significant (*P* > 0.05).

On the contrary, bacterial community composition was significantly different between animals when measured by analysis of similarities (ANOSIM) of unweighted UniFrac distance (*P* < 0.001). The unweighted UniFrac principal coordinate analysis (PCoA) plot (Figure 5C) visually confirmed the distinct separation of microbial communities among different ages of piglets. The first group of samples in the upper left quadrant were from 0-d piglets, which were more dispersed than the other age groups. The second cluster in the top-right quadrant of the PCoA chart consists of a sample of 42-d piglets. The third cluster in the middle of the four quadrants comprises samples from 7- to 35-d piglets clustered together but with a relatively low degree of aggregation. Overall, these results suggested that the β-diversity of the colonic digesta microbiota of piglets increased with age.

**Figure 6:**
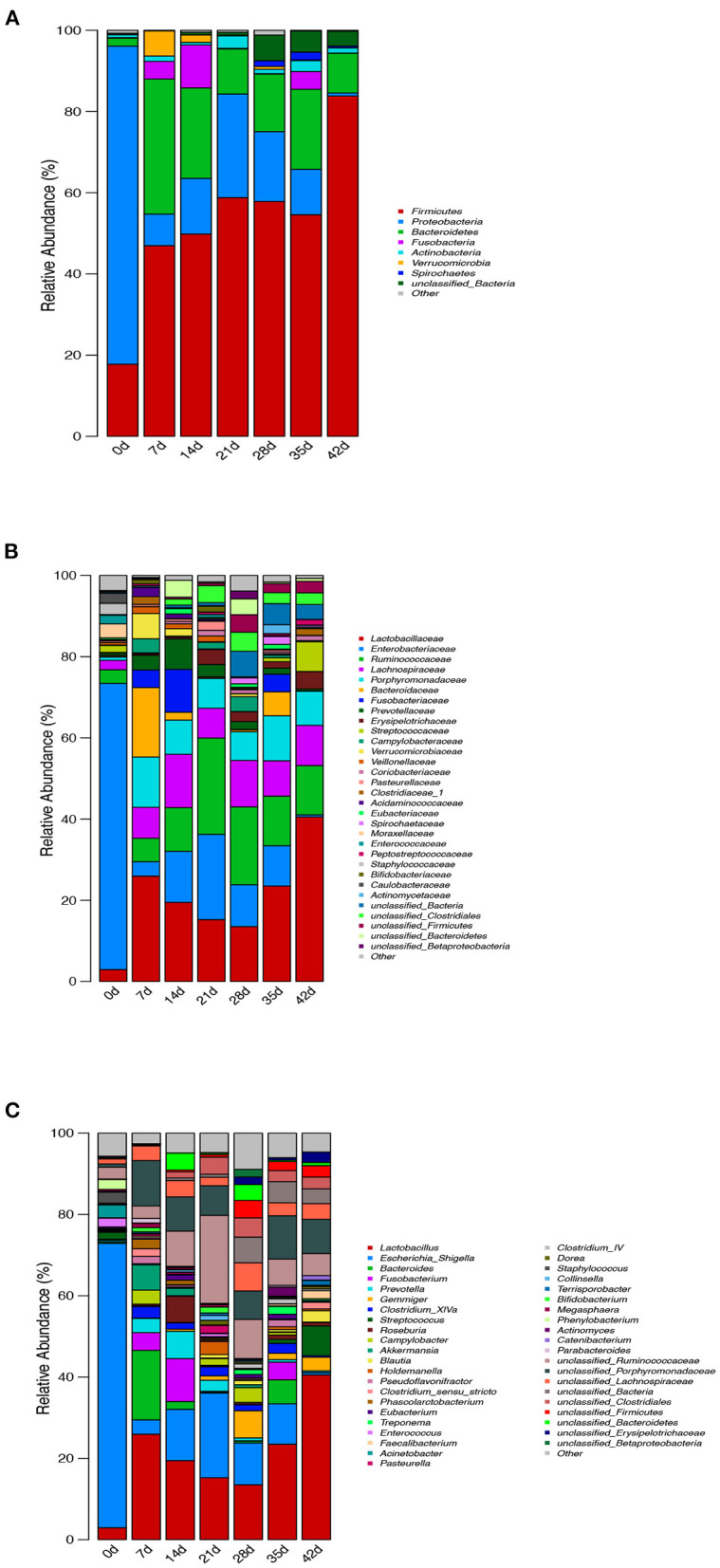
Effect of piglets' age on taxonomic classification of the 16S rRNA gene sequences at **(A)** phylum, **(B)** family, and **(C)** genus levels.

The relative abundance of intestinal microflora at phylum, family, and genus levels of piglets at different ages is shown in Figure 6. At the phylum level, the seven growth stage groups are mainly composed of seven phyla as follows: *Firmicutes, Proteobacteria, Bacteroidetes, Fusobacteria, Actinobacteria, Verrucomicrobia*, and *Spirochaetes*. *Firmicutes* (*P* = 0.001), *Proteobacteria* (*P* < 0.001), and *Bacteroidetes* (*P* = 0.003) were the most dominant among the seven phyla in the samples, regardless of age, and comprised more than 85% of the total sequences. The bacterial abundances of distinct phyla differed in the seven groups. *Firmicutes* (*P* = 0.001) was the most predominant phylum after birth (7–42 d), accounting for more than 46% of the sequences, while *Proteobacteria* (*P* < 0.001) was the main phylum of newborn piglets (0 d), accounting for more than 78% of the sequence. At 42 d, a higher percentage (83%) of the sequences was assigned to *Firmicutes*, but there was no significant difference from 7 to 35 d. *Bacteroidetes* and *Proteobacteria* were the second largest phylum at 7 d (33%), 14 d (22%), 35 d (20%), 42 d (10%), 0 d (78%), 21 d (26%), and 28 d (17%), respectively (Figure 6A).

Surprisingly, at the family level, *Enterobacteriaceae* of newborn piglets was the most abundant bacterial family, accounting for ~70% of the sequences. The abundance of *Enterobacteriaceae* decreased gradually during lactation, increased after weaning, and then decreased gradually from 28 to 42 d, but the change in *Lactobacillaceae* was the opposite. In addition, weaning increased the abundance of *Ruminococcaceae* compared with lactation and post-weaning. When comparing the microbiota during lactation and after weaning, there were significant differences. First, *Prevotellaceae* decreased nearly 4-fold from an average of 4.7% during lactation to 1.3% during weaning. This coincided with a decrease in the population of *Bacteroidaceae* and *Fusobacteriaceae* from 6.4 and 5.0% during lactation to 2.1 and 1.5% during weaning, respectively (on average). There is also an increase in *Streptococcaceae* from 0.2 to 2.8% after weaning. *Lactobacillaceae* increased over time from 13.5 to 40.5% after weaning. In contrast, *Lachnospiraceae, Porphyromonadaceae*, and *Campylobacteraceae* did not change dramatically throughout the study (Figure 6B).

To further investigate the taxonomic compositions of piglets at different days of age, a total of 281 genera were identified from the bacterial community of colonic digesta of piglets. Among these genera identified, 13 abundant genera were detected, which contained more than 5% of the total sequence in at least one sample. The 13 abundant genera were as follows: *Lactobacillus, Escherichia_Shigella, Bacteroides, Fusobacterium, Prevotella, Gemmiger, Streptococcus, Roseburia, Akkermansia, unclassified_Ruminococcaceae, unclassified_Porphyromonadaceae, unclassified_Lachnospiraceae*, and *unclassified_Bacteria* (Figure 6C). All the 13 abundant genera plus the unclassified genera accounted for over 62% of the total sequences in the samples, regardless of the age of piglets. Genus *Escherichia_Shigella* belonged to phylum *Proteobacteria* and had the highest abundance at 0 and 21 days of age, while *Lactobacillus* belonged to phylum *Firmicutes* and had the highest abundance at 7–42 d. With the increase in piglets' age, the abundance of *Escherichia_Shigella* and *unclassified_Ruminococcaceae* decreased, but increased at weaning, while the change in *Lactobacillus* was the opposite. Similarly, the abundance of *Prevotella, Roseburia, Akkermansia, Pasteurella, Bifidobacterium*, and *Megasphaera* was higher during lactation than after weaning, while the change in *Gemmiger, Blautia, Treponema, Faecalibacterium, Clostridium_IV*, and *unclassified_Bacteria* was opposite.

### Microbiota–Metabolite Correlation

**Figure 7:**
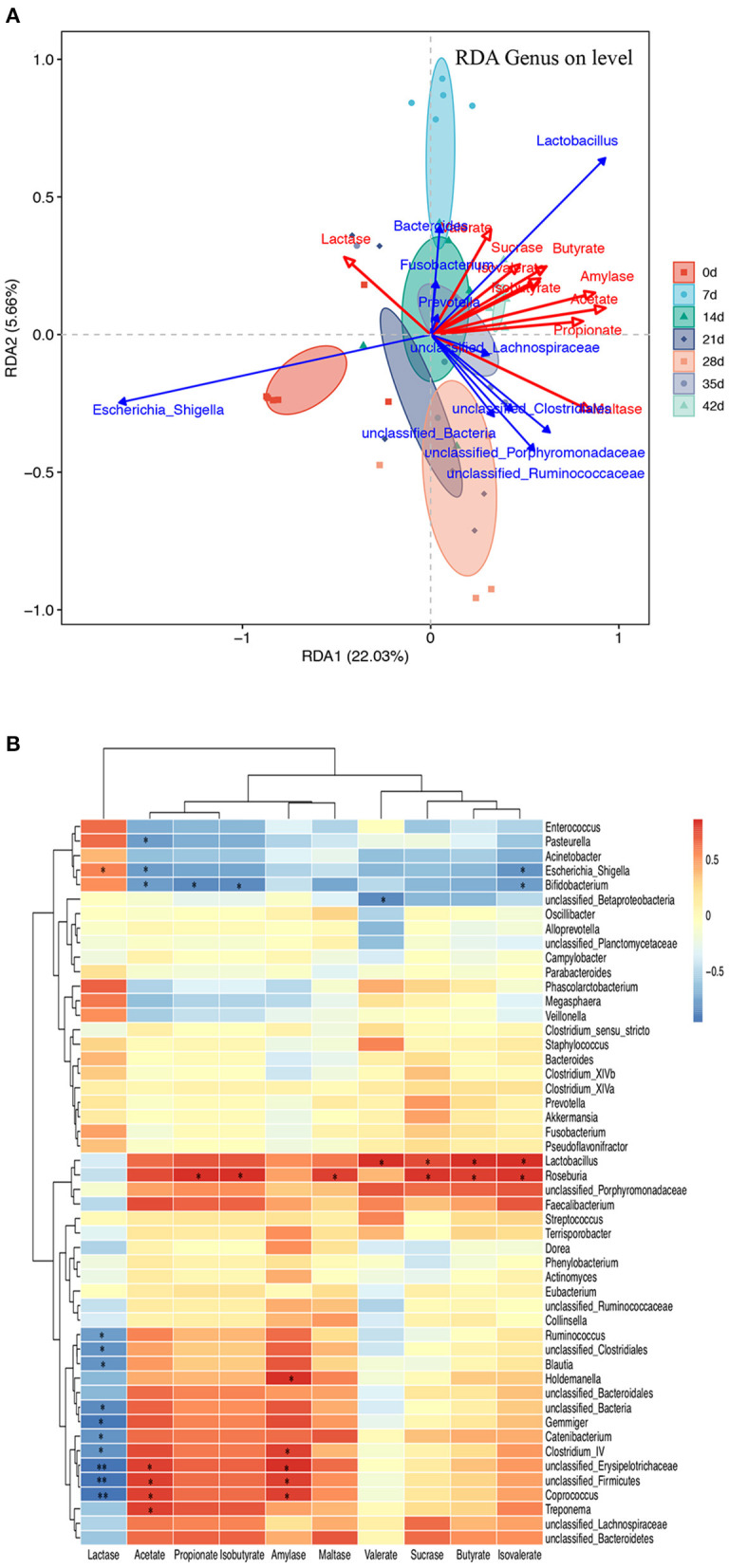
Microbiota–metabolite correlation. **(A)** Triplot of RDA of the colonic microbial composition at genus level relative to pancreatic amylase, jejunal disaccharidase, and colonic SCFAs. The microbiota of different age groups are represented by different colors. Constrained explanatory variables (amylase, SCFA, and disaccharidase) are indicated by red arrows. Responding taxa are indicated by blue arrows, and only those with a higher fit in the ordination plot are labeled. The first (22.03% interpretation) and second coordinates (5.66% interpretation) are plotted. **(B)** A heatmap of the correlation analysis was conducted between the top 50 bacterial genera and the environmental factors. ^*^0.01 < *P* ≤ 0.05 and ^**^0.001 < *P* ≤ 0.01.

The triplot of redundancy analysis (RDA) was conducted based on genus-level microorganisms and their environmental factors (amylase, SCFAs, and disaccharidase, Figure 7A), indicating that piglets of different ages were separated on the first constraint axis. RDA indicated that there were positive correlations among SCFAs, amylase, and sucrase and a negative correlation between lactase and SCFAs. In addition, a correlation between the top 50 microbial genera and the environmental factors was determined by calculating Spearman's correlation coefficients and was directly reflected by a heatmap (Figure 7B). The threshold |*R*| >0.4 is considered relevant. The results indicated that *Lactobacillus, unclassified_Ruminococcaceae*, and *Faecalibacterium* were positively correlated with amylase and SCFAs, while *unclassified_Lachnospiraceae, unclassified_Bacteroidetes*, and *Roseburia* were positively correlated with sucrase and maltase. *Escherichia_Shigella, Fusobacterium*, and *Phascolarctobacterium* were positively correlated with lactase, while *unclassified_Bacteria, unclassified_Clostridiale*s, *Gemmiger*, and *unclassified_Firmicutes* were negatively correlated with lactase. *Unclassified_Clostridiales* was positively correlated with acetate and negatively correlated with valerate. *Lactobacillus, unclassified_Bacteroidetes*, and *Roseburia* were positively correlated with amylase, sucrase, and maltase, while *Escherichia_Shigella* and *Bifidobacterium* were negatively correlated with them.

## Discussion

The first goal of this study was to better understand the changes in the digestive function of piglets from birth to post-weaning. So far, under the background that early weaning models are widely used, there are few studies on the changes in the intestinal digestive function with age in piglets (Corring et al., 1978; Owsley et al., 1986; Hedemann et al., 2003). Therefore, there are differences between the early research and the current situation of piglets, and new research is needed to provide a theoretical basis to guide production.

The intestine is the main place for digestion and absorption of nutrients, and the early postnatal period is a key time for intestinal development. The tissue mass and absorption surface area of the small intestine of newborn piglets increased significantly, for example, the number of mucosal cells increased by 50% on the first day after birth and doubled on the third day after birth (Widdowson and Crabb, 1976; Xu et al., 1992). These studies show that the intestine develops most rapidly in the early stages of piglets. In this study, our measurement of intestinal tissue characteristics is basically consistent with the data in the literature, which confirms that the animals used in this study are healthy and normal (Skrzypek et al., 2018; Verdile et al., 2019). Research showed that the morphological structure of the duodenum will change with the growth of piglets during lactation, such as the increase in villus height, crypt depth, and intestinal wall thickness (Wiyaporn et al., 2013). For instance, the villus height of the small intestine of piglets increases by 33% in the first week after birth and reaches its maximum in the second week after birth (Skrzypek et al., 2005). Consistent with previous studies, duodenal villus height increased continuously from 0 to 14 d, peaked at 14 d, decreased at weaning (21–28 d), and then increased at 35–42 d. However, the changing trend of jejunum and ileum is inconsistent with that of the duodenum, and the maximum value appeared at 7 and 0 d, respectively. One plausible explanation for the inconsistent peak time of villus height may be that the development of the small intestine structure is basically completed and the villus height is high at birth. Therefore, through this experiment, we once again demonstrated that the structure and digestive function of small intestine of piglets began from prenatal.

At birth, the small intestine is almost complete and digestion can begin after the first intake of colostrum. The integrity of intestinal development will affect the utilization efficiency of carbohydrates because it will affect the disaccharidase activity of piglets (Tsukahara et al., 2016). Since the pancreas is an important organ involved in nutrient digestion, in order to further understand the changes in the digestive ability of piglets with the increase in age, we measured the digestive enzymes secreted by the pancreas. Our results showed that the activities of sucrase and maltase in the intestine and amylase in the pancreas increased with age during lactation, decreased during weaning, and then increased during nursery, while the activities of lactase continued to decrease with age. The activities of lipase, trypsin, and chymotrypsin decreased with age during lactation and weaning but increased during the nursery. These results are consistent with previous studies (Bellinge et al., 2005; Ito et al., 2019). In addition, sucrase and maltase activities are important markers for evaluating intestinal development, and the activity intensity of pancreatic enzymes is an indicator to measure digestive ability (Huygelen et al., 2015; Pieper et al., 2016; Yuan et al., 2017). Thus, increased sucrase, maltase, and pancreatic enzyme activities imply rapid maturation of the small intestine and digestive capacity, respectively. Furthermore, higher lactase activity during lactation and higher sucrase and maltase activities during nursery were helpful to degrade polysaccharides in sow milk and feed into monosaccharides, respectively. This facilitates the absorption and utilization of carbohydrates by the body, thereby promoting intestinal maturation and host growth. Overall, these results suggest that the digestive capacity of the intestine and pancreas increases with age, and the increased carbohydrate degradation rate in the diet promotes the digestion and absorption of nutrients by increasing the activity of amylase and disaccharidase.

This study also measured short-chain fatty acids in the hindgut, which are key metabolites interacting with the intestinal microbiota and can affect intestinal health and systemic metabolism (Yao et al., 2020; Zhou et al., 2020). Our results showed that the concentration of SCFAs increased with age but decreased at weaning. Studies have confirmed that higher concentrations of SCFAs in the intestine contribute to better growth performance (Le Gall et al., 2009). In addition, butyrate has been shown to have positive effects on pathogen control and intestinal barrier function, particularly energy utilization in the colon, gastrointestinal cell proliferation, and pH stabilization in the intestinal lumen (Guilloteau et al., 2010; Kelly et al., 2015).

The mammalian gastrointestinal tract contains a wide variety and active microbial community that serves as an important barrier against pathogens and plays an integral role in promoting the development of the intestinal immune system and maintaining normal intestinal function (Buffie and Pamer, 2013; Kamada et al., 2013). The present study shows a significantly increased α-diversity in the intestinal bacterial community with the age of piglets but decreased at the weaning stage. Moreover, recent studies showed that the α-diversity of intestinal bacteria increased significantly with the time interval of ~1 month after weaning (Niu et al., 2015; Zhao et al., 2015). One of the most striking observations in this study was that with the exception of *Firmicutes* and *Proteobacteria*, which were the two most dominant phyla in the piglet intestinal microbiota at 0 and 21 d, the predominant phylum in the other groups was *Firmicutes* and *Bacteroidetes*. Our results are consistent with the finding of Zhao et al. which indicated the maximum abundance of *Proteobacteria* at birth and then it begins to decline (Zhao et al., 2015). In this study, *Escherichia_Shigella* was the most abundant bacteria in the intestinal of newborn piglets and subsequently, its abundance decreased during lactation and increased again during weaning. Piglet diarrhea can be attributed to the presence of certain bacteria in the microbiota, such as *Escherichia_Shigella* (Schokker et al., 2015; Hu et al., 2016). This result suggests that pathogenic species are often present in the gastrointestinal tract of infants or early piglets, waiting for a potential opportunity to become pathogens. The results of this study also showed that *Lactobacillus* belonging to the phylum *Firmicutes* was the most abundant genera in intestinal bacterial communities during lactation and nursery periods. It is well known that *Lactobacillus* will produce acetate, and the increase in *Lactobacillus* abundance will increase the concentration of acetate in the hindgut, which is consistent with our previous results on SCFAs. Furthermore, *Lactobacillus* and *Escherichia_Shigella* are the core microbiota in pre-weaning piglets, suggesting that they play a key role in the establishment and maintenance of the postnatal intestinal microbiota in piglets. In addition, the abundance of *Prevotella* increased with age during lactation, which is in line with the results of the study based on the intestinal microbiota of infants (from newborn to 12 months) (Bäckhed et al., 2015). *Prevotella* is a key microbe member of the animal gastrointestinal tract, and it is not only important for the degradation of starch and plant polysaccharides but also has strong catabolism of mucin (Kovatcheva-Datchary et al., 2015; Fang et al., 2017).

Using RDA, amylase, SCFAs, and disaccharidase were significantly associated with colonic microbiota. Furthermore, Spearman's correlation analysis of the top 50 microbiota genera and hindgut environmental factors showed that the microbiota bacteria with positive effects on SCFAs were *Lactobacillus, unclassified_Porphyromonadaceae*, and *Faecalibacterium*, while the microbiota negatively correlated with SCFAs were *Escherichia_Shigella, Acinetobacter*, and *Bifidobacterium*. Previous studies have found that *Lactobacillus* bacteria promote early piglet development and improve the intestinal health of newborn piglets by modulating intestinal microbiota (Dowarah et al., 2017; Yang et al., 2018; Zhang et al., 2019). The abundance of *Bifidobacteria* decreased with increasing age. As *Bifidobacteria* have been proved to inhibit the inflammatory response of intestinal epithelial cells, the decrease in its relative abundance with age may be related to the increase in chronic inflammation in the elderly. In addition, *Faecalibacterium* is a butyrate-producing bacterium, which increases with age. *Unclassified_Bacteroidetes* were positively correlated with amylase, sucrase, and maltase. *Bacteroidetes* are involved in nutrient metabolism, including carbohydrate fermentation, polysaccharides, and steroid metabolism, and are essential for maintaining the normal physiological function of the intestine (Yang et al., 2019).

Overall, our results suggested that the most significant difference between weaning, lactation, and the nursery was the decrease in the abundance of bacteria producing SCFAs and bacteria involved in carbohydrate metabolism. Many of the bacterial taxa that showed reduced numbers in piglets with diarrhea were related to the carbohydrate production of SCFAs, which was also observed in humans with Crohn's disease, suggesting that abnormal carbohydrate metabolism may be an important feature of diarrheal disease in piglets (Erickson et al., 2012). Therefore, we should further study the carbohydrate metabolism of piglets in future research.

## Conclusion

In this experiment, we explored the digestive function and microbial changes in piglets from birth to nursery and the correlation between intestinal microorganisms and digestive enzymes and metabolites. Based on the results, we infer that age and growth environment, including diet composition and weaning, are the key factors for the formation of piglet intestinal microbiota. Diarrhea caused by carbohydrate digestion deserves the attention of nutritionists. Supplementing bacteria conducive to carbohydrate digestion at an appropriate time will be beneficial to the health of piglets and infants. Our results broaden the understanding of the digestive function and microbial changes in piglets at different stages. These results will provide help for the experimental design of host–microbial interaction.

## Data Availability Statement

The datasets presented in this study can be found in online repositories. The names of the repository/repositories and accession number(s) can be found below: NCBI BioProject—PRJNA816983.

## Ethics Statement

The animal study was reviewed and approved by Institutional Animal Care Advisory Committee for Sichuan Agricultural University.

## Author Contributions

JH and DC designed the whole experiment. XG performed the experiment, including chemical analysis, statistical analysis, and manuscript writing. BY, ZH, and JY verified the validity of the experiment and checked the results. PZ, JL, and YL participated in the experimental design and provided valuable advice. All authors have read and approved the final version of the manuscript.

## Funding

This research was supported by grants from the National Natural Science Foundation of China (Project No. 31730091).

## Conflict of Interest

The authors declare that the research was conducted in the absence of any commercial or financial relationships that could be construed as a potential conflict of interest.

## Publisher's Note

All claims expressed in this article are solely those of the authors and do not necessarily represent those of their affiliated organizations, or those of the publisher, the editors and the reviewers. Any product that may be evaluated in this article, or claim that may be made by its manufacturer, is not guaranteed or endorsed by the publisher.

## References

[B1] Bäckhed F. Roswall J. Peng Y. Feng Q. Jia H. Kovatcheva-Datchary P.. (2015). Dynamics and stabilization of the human gut microbiome during the first year of life. Cell Host Microbe. 17, 690–703. 10.1016/j.chom.2015.04.004 25974306

[B2] Bellinge R. Liberles D. Iaschi S. O'brien P. Tay G. (2005). Myostatin and its implications on animal breeding: a review. Anim. Genet. 36, 1–6. 10.1111/j.1365-2052.2004.01229.x 15670124

[B3] Buffie C. G. Pamer E. G. (2013). Microbiota-mediated colonization resistance against intestinal pathogens. Nat. Rev. Immunol. 13, 790–801. 10.1038/nri3535 24096337 PMC4194195

[B4] Caporaso J. G. Kuczynski J. Stombaugh J. Bittinger K. Bushman F. D. Costello E. K.. (2010). QIIME allows analysis of high-throughput community sequencing data. Nat. Methods. 7, 335–336. 10.1038/nmeth.f.303 20383131 PMC3156573

[B5] Corring T. Aumaitre A. Durand G. (1978). Development of digestive enzymes in the piglet from birth to 8 weeks. Ann. Nutr. Metab. 22, 231–243. 10.1159/000176219 634514

[B6] Cui Z. Dibley M. J. (2012). Trends in dietary energy, fat, carbohydrate and protein intake in Chinese children and adolescents from 1991 to 2009. Br. J. Nutr. 108, 1292–1299. 10.1017/S0007114511006891 22244308 PMC3488814

[B7] Dou S. Gadonna-Widehem P. Rome V. R. Hamoudi D. Rhazi L.. (2017). Characterisation of early-life fecal microbiota in susceptible and healthy pigs to post-weaning diarrhoea. PLoS ONE. 12, e0169851. 10.1371/journal.pone.0169851 28072880 PMC5225014

[B8] Dowarah R. Verma A. Agarwal N. Patel B. Singh P. (2017). Effect of swine based probiotic on performance, diarrhoea scores, intestinal microbiota and gut health of grower-finisher crossbred pigs. Livestock Sci. 195, 74–79. 10.1016/j.livsci.2016.11.006

[B9] Erickson A. R. Cantarel B. L. Lamendella R. Darzi Y. Mongodin E. F. Pan C.. (2012). Integrated metagenomics/metaproteomics reveals human host-microbiota signatures of Crohn's disease. PLoS ONE. 7, e49138. 10.1371/journal.pone.0049138 23209564 PMC3509130

[B10] Fang S. Xiong X. Su Y. Huang L. Chen C. (2017). 16S rRNA gene-based association study identified microbial taxa associated with pork intramuscular fat content in feces and cecum lumen. BMC Microbiology. 17, 162. 10.1186/s12866-017-1055-x 28724349 PMC5518119

[B11] Green G. Brostoff J. Hudspith B. Michael M. Mylonaki M. Rayment N.. (2006). Molecular characterization of the bacteria adherent to human colorectal mucosa. J. Appl. Microbiol. 100, 460–469. 10.1111/j.1365-2672.2005.02783.x 16478485

[B12] Guilloteau P. Martin L. Eeckhaut V. Ducatelle R. Zabielski R. Van Immerseel F. (2010). From the gut to the peripheral tissues: the multiple effects of butyrate. Nutr. Res. Rev. 23, 366–384. 10.1017/S0954422410000247 20937167

[B13] Hedemann M. S. Højsgaard S. Jensen B. B. (2003). Small intestinal morphology and activity of intestinal peptidases in piglets around weaning. J. Anim. Physiol. Anim. Nutr. 87, 32–41. 10.1046/j.1439-0396.2003.00405.x 14511147

[B14] Hu J. Nie Y. Chen J. Zhang Y. Wang Z. Fan Q.. (2016). Gradual changes of gut microbiota in weaned miniature piglets. Front. Microbiol. 7, 1727. 10.3389/fmicb.2016.01727 27853453 PMC5090779

[B15] Huygelen V. De Vos M. Prims S. Vergauwen H. Fransen E. Casteleyn C.. (2015). Birth weight has no influence on the morphology, digestive capacity and motility of the small intestine in suckling pigs. Livestock Sci. 182, 129–136. 10.1016/j.livsci.2015.11.003

[B16] Ito K. Matsuura K. Mihara Y. Sakamoto Y. Hasegawa K. Kokudo N.. (2019). Delivery of pancreatic digestive enzymes into the gastrointestinal tract by pancreatic exocrine tissue transplant. Sci. Rep. 9, 1–11. 10.1038/s41598-019-42362-z 30976035 PMC6459827

[B17] Kamada N. Seo S.-U. Chen G. Y. Núñez G. (2013). Role of the gut microbiota in immunity and inflammatory disease. Nat. Rev. Immunol. 13, 321–335. 10.1038/nri3430 23618829

[B18] Kelly C. J. Zheng L. Campbell E. L. Saeedi B. Colgan S. P. (2015). Crosstalk between microbiota-derived short-chain fatty acids and intestinal epithelial HIF augments tissue barrier function. Cell Host Microbe. 17, 662–671. 10.1016/j.chom.2015.03.005 25865369 PMC4433427

[B19] Kim H. B. Borewicz K. White B. A. Singer R. S. Sreevatsan S. Tu Z. J.. (2012). Microbial shifts in the swine distal gut in response to the treatment with antimicrobial growth promoter, tylosin. Proc. Natl. Acad. Sci. 109, 15485–15490. 10.1073/pnas.1205147109 22955886 PMC3458334

[B20] Kovatcheva-Datchary P. Nilsson A. Akrami R. Lee Y. S. De Vadder F. Arora T.. (2015). Dietary fiber-induced improvement in glucose metabolism is associated with increased abundance of Prevotella. Cell Metab. 22, 971–982. 10.1016/j.cmet.2015.10.001 26552345

[B21] Krajmalnik-Brown R. Ilhan Z.-E. Kang D.-W. DiBaise J. K. (2012). Effects of gut microbes on nutrient absorption and energy regulation. Nutr. Clin. Pract. 27, 201–214. 10.1177/0884533611436116 22367888 PMC3601187

[B22] Le Gall M. Gallois M. Seve B. Louveau I. Holst J. J. Oswald I. P.. (2009). Comparative effect of orally administered sodium butyrate before or after weaning on growth and several indices of gastrointestinal biology of piglets. Br. J. Nutr. 102, 1285–1296. 10.1017/S0007114509990213 19480733

[B23] Lin A. H.-M. (2018). Structure and digestion of common complementary food starches. J. Pediatr. Gastroenterol. Nutr. 66, S35–S38. 10.1097/MPG.0000000000001973 29762374

[B24] Niu Q. Li P. Hao S. Zhang Y. Kim S. W. Li H.. (2015). Dynamic distribution of the gut microbiota and the relationship with apparent crude fiber digestibility and growth stages in pigs. Sci. Rep. 5, 1–7. 10.1038/srep09938 25898122 PMC4404679

[B25] NRC (2012). Nutrient Requirements of Swine, 11th Edn. Washington, DC: Natl Acad Ress.

[B26] Owsley W. Orr D. Jr. Tribble L. (1986). Effects of age and diet on the development of the pancreas and the synthesis and secretion of pancreatic enzymes in the young pig. J. Anim. Sci. 63, 497–504. 10.2527/jas1986.632497x 2428799

[B27] Pieper R. Scharek-Tedin L. Zetzsche A. Röhe I. Kröger S. Vahjen W.. (2016). Bovine milk–based formula leads to early maturation-like morphological, immunological, and functional changes in the jejunum of neonatal piglets. J. Anim. Sci. 94, 989–999. 10.2527/jas.2015-9942 27065261

[B28] Pluske J. Williams I. Aherne F. (1996). Maintenance of villous height and crypt depth in piglets by providing continuous nutrition after weaning. Anim. Sci. 62, 131–144. 10.1017/S1357729800014417

[B29] Porter M. Murray R. (2001). The volatility of components of grass silage on oven drying and the inter-relationship between dry-matter content estimated by different analytical methods. Grass Forage Sci. 56, 405–411. 10.1046/j.1365-2494.2001.00292.x

[B30] Ramayo-Caldas Y. Mach N. Lepage P. Levenez F. Denis C. Lemonnier G.. (2016). Phylogenetic network analysis applied to pig gut microbiota identifies an ecosystem structure linked with growth traits. ISME J. 10, 2973–2977. 10.1038/ismej.2016.77 27177190 PMC5148198

[B31] Schokker D. Zhang J. Vastenhouw S. A. Heilig H. G. Smidt H. Rebel J. M.. (2015). Long-lasting effects of early-life antibiotic treatment and routine animal handling on gut microbiota composition and immune system in pigs. PLoS ONE. 10, e0116523. 10.1371/journal.pone.0116523 25658611 PMC4319779

[B32] Shulman R. J. (2018). Starch malabsorption in infants. J. Pediatr. Gastroenterol. Nutr. 66, S65–S67. 10.1097/MPG.0000000000001856 29762382

[B33] Skrzypek T. Kazimierczak W. Skrzypek H. Valverde Piedra J. Godlewski M. Zabielski R. (2018). Mechanisms involved in the development of the small intestine mucosal layer in postnatal piglets. J. Physiol. Pharmacol. 69, 127–138. 10.26402/jpp.2018.1.14 29769429

[B34] Skrzypek T. Piedra J. V. Skrzypek H. Wolinski J. Kazimierczak W. Szymanczyk S.. (2005). Light and scanning electron microscopy evaluation of the postnatal small intestinal mucosa development in pigs. J. Physiol. Pharmacol. 56, 71–87. 16077196

[B35] Tsukahara T. Inoue R. Nakatani M. Fukuta K. Kishino E. Ito T.. (2016). Influence of weaning age on the villous height and disaccharidase activities in the porcine small intestine. Anim. Sci. J. 87, 67–75. 10.1111/asj.12399 26153481

[B36] Verdile N. Mirmahmoudi R. Brevini T. Gandolfi F. (2019). Evolution of pig intestinal stem cells from birth to weaning. Animal. 13, 2830–2839. 10.1017/S1751731119001319 31199215

[B37] Widdowson E. Crabb D. (1976). Changes in the organs of pigs in response to feeding for the first 24 h after birth. Neonatology. 28, 261–271. 10.1159/000240827 1276297

[B38] Wiyaporn M. Thongsong B. Kalandakanond-Thongsong S. (2013). Growth and small intestine histomorphology of low and normal birth weight piglets during the early suckling period. Livestock Sci. 158, 215–222. 10.1016/j.livsci.2013.10.016

[B39] Xiong X. Tan B. Song M. Ji P. Kim K. Yin Y.. (2019). Nutritional intervention for the intestinal development and health of weaned pigs. Front. Vet. Sci. 6, 46. 10.3389/fvets.2019.00046 30847348 PMC6393345

[B40] Xu R. Mellor D. Tungthanathanich P. Birtles M. Reynolds G. Simpson H. (1992). Growth and morphological changes in the small and the large intestine in piglets during the first three days after birth. J. Dev. Physiol. 18, 161–172. 1284564

[B41] Yang J. Qian K. Wang C. Wu Y. (2018). Roles of probiotic lactobacilli inclusion in helping piglets establish healthy intestinal inter-environment for pathogen defense. Prob. Antimicrob. Proteins. 10, 243–250. 10.1007/s12602-017-9273-y 28361445

[B42] Yang Q. Huang X. Wang P. Yan Z. Sun W. Zhao S.. (2019). Longitudinal development of the gut microbiota in healthy and diarrheic piglets induced by age-related dietary changes. Microbiologyopen. 8, e923. 10.1002/mbo3.923 31496126 PMC6925166

[B43] Yao Y. Yan L. Chen H. Wu N. Wang W. Wang D. (2020). Cyclocarya paliurus polysaccharides alleviate type 2 diabetic symptoms by modulating gut microbiota and short-chain fatty acids. Phytomedicine. 77, 153268. 10.1016/j.phymed.2020.153268 32663709

[B44] Yatsunenko T. Rey F. E. Manary M. J. Trehan I. Dominguez-Bello M. G. Contreras M.. (2012). Human gut microbiome viewed across age and geography. Nature. 486, 222–227. 10.1038/nature11053 22699611 PMC3376388

[B45] Yuan L. Wang M. Zhang X. Wang Z. (2017). Effects of protease and non-starch polysaccharide enzyme on performance, digestive function, activity and gene expression of endogenous enzyme of broilers. PLoS ONE. 12, e0173941. 10.1371/journal.pone.0173941 28323908 PMC5360255

[B46] Zhang D. Liu H. Wang S. Zhang W. Wang J. Tian H.. (2019). Fecal microbiota and its correlation with fatty acids and free amino acids metabolism in piglets after a Lactobacillus strain oral administration. Front. Microbiol. 10, 785. 10.3389/fmicb.2019.00785 31040835 PMC6476935

[B47] Zhao W. Wang Y. Liu S. Huang J. Zhai Z. He C.. (2015). The dynamic distribution of porcine microbiota across different ages and gastrointestinal tract segments. PLoS ONE. 10, e0117441. 10.1371/journal.pone.0117441 25688558 PMC4331431

[B48] Zhou C. Li L. Li T. Sun L. Yin J. Guan H.. (2020). SCFAs induce autophagy in intestinal epithelial cells and relieve colitis by stabilizing HIF-1α. J. Mol. Med. 98, 1189–1202. 10.1007/s00109-020-01947-2 32696223

