# Supplementary material for: Developmental Profiling of Dietary Carbohydrate Digestion in Piglets
Source: Front Microbiol. 2022 Apr 29;13:896660. doi: 10.3389/fmicb.2022.896660 (PMC9100932; doi:10.3389/fmicb.2022.896660)
Supplement: Supplementary file 1 [file Table_1.DOCX]

Supplemental Table 1 Effect of piglet age on intestinal disaccharidase activity

| Item | 0d | 7d | 14d | 21d | 28d | 35d | 42d | Pooled SEM | *P-value* |
| --- | --- | --- | --- | --- | --- | --- | --- | --- | --- |
| Duodenum (U/mgprot) |  |  |  |  |  |  |  |  |  |
| Sucrase | 14.41^b^ | 14.19^b^ | 22.94^b^ | 12.64^b^ | 18.68^b^ | 24.64^b^ | 54.92^a^ | 2.64 | <0.001 |
| Maltase | 5.21^bc^ | 6.81^abc^ | 5.35^bc^ | 4.94^bc^ | 3.32^c^ | 11.40^a^ | 10.31^ab^ | 0.75 | 0.022 |
| Lactase | 158.75^a^ | 125.74^ab^ | 97.31^bc^ | 78.97^c^ | 21.46^d^ | 17.70^d^ | 18.23^d^ | 9.55 | <0.001 |
| Jejunum (U/mgprot) |  |  |  |  |  |  |  |  |  |
| Sucrase | 11.90 | 23.98 | 25.50 | 16.68 | 17.00 | 20.49 | 24.08 | 1.96 | 0.509 |
| Maltase | 6.04^c^ | 15.43^bc^ | 31.11^ab^ | 28.21^ab^ | 26.03^ab^ | 30.12^ab^ | 31.45^a^ | 2.23 | 0.006 |
| Lactase | 146.57^a^ | 123.07^a^ | 105.99^ab^ | 60.32^bc^ | 35.47^c^ | 41.29^c^ | 31.01^c^ | 9.09 | <0.001 |
| Ileum(U/mgprot) |  |  |  |  |  |  |  |  |  |
| Sucrase | 15.71^c^ | 16.83^c^ | 27.51^bc^ | 39.65^abc^ | 25.99^bc^ | 48.86^ab^ | 61.93^a^ | 4.35 | 0.012 |
| Maltase | 13.90^b^ | 38.30^a^ | 52.23^a^ | 35.54^a^ | 37.65^a^ | 48.69^a^ | 51.36^a^ | 2.94 | 0.002 |
| Lactase | 174.65^a^ | 101.26^b^ | 107.40^b^ | 49.21^c^ | 30.55^c^ | 30.68^c^ | 27.95^c^ | 9.73 | <0.001 |

Mean values with their standard errors

^a,b,c d^ Mean values within a row with different superscript letters were significantly different (*P* < 0.05)

Supplemental Table 2 Effect of piglet age on pancreatic digestive enzyme activity

| Item | 0d | 7d | 14d | 21d | 28d | 35d | 42d | Pooled SEM | *P-value* |
| --- | --- | --- | --- | --- | --- | --- | --- | --- | --- |
| Amylase (U/mgprot) | 47.20^c^ | 252.64^b^ | 255.89^b^ | 314.83^b^ | 263.05^b^ | 354.68^ab^ | 468.92^a^ | 26.46 | <0.001 |
| Lipase (U/gprot) | 33.59^bc^ | 25.26^cd^ | 20.30^cd^ | 14.57^d^ | 38.77^bc^ | 47.56^b^ | 86.65^a^ | 4.13 | <0.001 |
| Trypsin (U/mgprot) | 264.49^a^ | 181.33^abc^ | 178.86^bc^ | 105.99^c^ | 156.79^bc^ | 181.41^abc^ | 215.02^ab^ | 11.70 | 0.018 |
| Chymotrypsin (U/gprot) | 441.47^a^ | 425.87^a^ | 384.96^a^ | 177.77^b^ | 333.83^a^ | 305.83^ab^ | 346.65^a^ | 21.34 | 0.010 |

Mean values with their standard errors

^a,b,c^ Mean values within a row with different superscript letters were significantly different (*P* < 0.05)
